# 

**Published:** 1953

**Authors:** 


### THE MEDICAL JOURNAL OF THE SOUTH-WEST

*Incorporating*
*The Bristol Medico-Chirurgical Journal*

PUBLISHED UNDER THE AUSPICES OF THE
BRISTOL MEDICO-CHIRURGICAL SOCIETY

*Editor:*

J. E. CATES
M.D., M.R.C.P.

*with whom are associated*

A. H. GALE, M.D., D.P.H.: *Assistant Editor*
J. MACRAE, M.D., D.P.H., F.R.E.P.S.
N. J. BROWN, M.R.C.P.
J. P. PARTRIDGE, F.R.C.S.

*1953 - 1955 Vol. 70*

J. W. ARROWSMITH LTD., WINTERSTOKE ROAD, BRISTOL

WI
ME359
V. 70-71
1953/55-1956
c.1

### 1955 Contents of Vol. 70

page

Foreword. By Sir Philip Morris, c.b.e., m.a., ll.d. . . . . 1

"Marasmus". By A. V. Neale, m.d., f.r.c.p., d.p.h. . . . . 3

Underwater Breathing. By Ronald Woolmer, f.f.a.r.c.s. . . . 9

Medicine at Home and Abroad. By John Apley, m.d., m.r.c.p. . . 16

The Elderly Chronic Sick . . . . . . . . 20

Observations on Blindness. By R. Ramsay Garden, m.a., m.b., ch.b., d.o.m.s. . 33

Diffuse Angiomatosis of the Small Intestine Causing Melaena. By John A. Vere Nicoll, f.r.c.s., d.a. . . . . . . . 47

Phaeochromocytoma of Adrenal Gland with Paroxysmal Hypertension. By G. M. Colson, b.m., m.r.c.p . . . . . . . . 50

Herpes Zoster Encephalitis. By R. D. G. Creery, m.d., m.r.c.p., d.c.h. . 54

Herpes Zoster Generalisatus. By Mark Hewitt, m.b., m.r.c.p. . . 57

College of General Practitioners, South Western Regional Faculty . . 61

Damned Drugs (Part I). By G. E. F. Sutton, *M.C.*, m.d., f.r.c.p. . . 71

Practical Points in the Home Treatment of Tuberculosis. By C. de W. Kitcat, m.r.c.s., l.r.c.p. . . . . . . . . . 79

The Tropics on our Doorstep. By Sir George R. McRobert, *C.I.E.*, m.d., f.r.c.p. . . . . . . . . . 83

Colonial Medical Research. By H. S. L. Heller, m.d., ph.d., m.r.c.p. . . 91

A Radiologist Visits Africa. By J. H. Middlemiss, m.d., f.f.r., d.m.r.d. . 95

Mongolism, Hypertrophic Pyloric Stenosis Treated with "Skopyl", and Death from Miliary Tuberculosis and Meningitis. (A Clinico-Pathological Report). 97

Book Review . . . . . . . . . 102

Damned Drugs (Part II). By G. E. F. Sutton, *M.C.*, m.d., f.r.c.p. . . 103

Cat - Scratch Disease. By D. Bryant, m.b., b.chir., and R. P. Warin, m.d., m.r.c.p. . . . . . . . . . 113

Some New Methods of Radiotherapy. By R. C. Tudway, m.b., b.s., b.sc., f.f.r. . 116

"General Practice". By G. F. Abercrombie, v.r.d., m.d. . . . 120

Colloid Cyst of Third Ventricle (A Clinico-Pathological Report). . . 127

Cousin Marriage. By J. A. Fraser Roberts, m.a., m.d., d.sc., f.r.c.p. . . 142

Meningitis in Childhood. By Beryl D. Corner, m.d., f.r.c.p. . . . 150

pa[illegible]

Meningitis. By A. M. G. Campbell, M.D., F.R.C.P. . . . . . 16[illegible]

What the G.P. Wants from a Casualty Department. By A General Practitioner 16[illegible]

Casualty Departments. By Herbert K. Bourns, M.B., F.R.C.S. . . . 16[illegible]

Jaundice with Hepatic Failure (A Clinico-Pathological Report) . . . 17[illegible]

That Backbone. By A. Gordon Heron, M.B., CH.B. . . . . . 18[illegible]

Home Care of the Diabetic. By C. T. Andrews, M.D., F.R.C.P. . . . 19[illegible]

Child Life in Past Times. By S. W. Hinds, M.D., M.R.C.P. . . . . 19[illegible]

The Changing Pattern of Pulmonary Tuberculosis. By R. A. Craig, M.D., B.SC., M.R.C.P. . . . . . . . . . . 20[illegible]

The General and Special in Medical Practice. By E. M. Bluestone, M.D. . 21[illegible]

Haemorrhage from a Cavernous Haemangioma of the Cord Clinically Resembling an Ascending Myelitis (A Clinico-Pathological Report) . . . 21[illegible]

Editorial Notes . . . . . . . . 31, 69, 14[illegible]

Annotations . . . . . . 20, 100, 130, 2[illegible]

Correspondence . . . . . . . . 61, 1[illegible]

News from Societies . . . . . . 20, 62, 131, 176, 2[illegible]

Appointments . . . . . 23, 65, 101, 138, 179, 2[illegible]

Notes and News . . . . . 27, 67, 100, 137, 178, 2[illegible]

3375 0
99-10

### THE MEDICAL JOURNAL OF THE SOUTH-WEST

*(Incorporating the Bristol Medico-Chirurgical Journal)*

VOL. 70 (i) 1953 NO. 253

### Contents

*PUBLISHED QUARTERLY*

*ANNUAL SUBSCRIPTION* **16/-** *POST FREE*

PUBLISHED UNDER THE AUSPICES OF THE

BRISTOL MEDICO-CHIRURGICAL SOCIETY

---

### Editorial Committee

MR. H. KEITH LUCAS, 60 St. John's Road, Bristol 8. *Editor.*

DR. J. E. CATES, Dept. of Medicine, Bristol Royal Infirmary } *Assistant Editors.*
DR. A. H. GALE, The University, Bristol 8 }

DR. J. MACRAE, Ham Green Hospital. *Treasurer.*

DR. A. M. MACLACHLAN, 167 Redland Road, Bristol 6.

DR. F. J. Y. WOOD, Dept. of Medicine, Bristol Royal Infirmary.

*Local Correspondents*

*Bath* . . Mr. A. D. BATEMAN, 3 The Circus, Bath.

*Exeter* . . Dr. L. N. JACKSON, Mount Jocelyn, Crediton, Devon.

*Gloucester* . Dr. R. F. JARRETT, 9 College Green, Gloucester.

*Plymouth* . Dr. C. R. CROFT, Lexden, Hartley Av., Plymouth.

*Taunton* . Dr. M. MCALLEY, 1 The Crescent, Taunton.

*Torquay* . Dr. A. ROBINSON THOMAS, 20 Devon Square, Newton Abbot, Devon.

*Truro* . . Dr. F. D. M. HOCKING, St. Andrews, Perranporth, Cornwall.

*Yeovil* . . Mr. J. A. VERE NICHOLL, Knapp House, Yeovil, Somerset.

### NOTICE TO CONTRIBUTORS

Original articles are invited, provided they have not been published elsewhere. Notes of forthcoming events, meetings held, degrees conferred, staff appointments, and other local news are welcomed. The editorial committee reserves the right to make literary corrections.

Illustrations should always be supplied ready for reproduction. All re-touchings or other operations required will be charged to the author. Line drawings should be drawn in Indian ink. We regret that we must ask authors to pay for making blocks, if this is necessary.

J. W. ARROWSMITH LTD., SMALL STREET,

BRISTOL.

### *Appointments*

### *UNIVERSITY OF BRISTOL AND UNITED BRISTOL HOSPITALS*

| *Name* | *Appointment* | *Formerly* |
|---|---|---|
| ANCILL, R. J., M.B. | Lecturer in Anatomy. | Demonstrator. |
| BAILY, R. A. J., M.B., F.R.C.S. | Senior Registrar in Orthopaedic Surgery. | Registrar, U.B.H. |
| BOLTON, F. G., M.B., M.R.C.P. | Senior Registrar in Pathology. | Registrar, U.B.H. |
| BOURNS, H. K., M.B., F.R.C.S. | Consultant Surgeon in Charge of Casualty Department. | Senior Registrar, Plymouth. |
| CANTRELL, G. R. M.B., D.O. | Registrar in Ophthalmology. | Royal Eye Hospital, Manchester. |
| CLUTTON-BROCK, J., M.B., F.F.A. | Consultant Anaesthetist and Lecturer in Anaesthetics. | Consultant Anaesthetist, Birmingham. |
| COLSON, G. M., B.M., M.R.C.P. | Senior Registrar in Medicine. | Senior Registrar, Exeter (Transfer). |
| DOWNIE, C. C., M.B. | Assistant Medical Officer, Student Health Service. | S.R.O., Bristol Royal Infirmary. |
| EDWARDS, E. M., M.B., M.R.C.O.G. | Registrar in Obstetrics and Gynaecology. | Redhill County Hospital. |
| FREEMAN, R. H., M.B., F.R.C.S. | Registrar in Orthopaedics. | Robert Jones and Agnes Hunt Hospital, Oswestry. |
| GUY, J., MISS, M.B., D.C.H. | Registrar in Pathology. | Demonstrator in Pathology, Bristol University. |
| HADFIELD, G. J., M.B., F.R.C.S. | Senior Registrar in Surgery. | Registrar in Surgery, United Bristol Hospitals. |
| HUDSON, G., B.SC., M.B. | Lecturer in Anatomy. | — |
| JOHNSON, D. H., M.D. | Registrar in Pathology. | — |

| *Name* | *Appointment* | *Formerly* |
|---|---|---|
| MacGregor, W. G., M.B., F.R.C.S., M.R.C.O.G. | Lecturer in Obstetrics and Gynaecology. | Senior Registrar, Bristol. |
| Meek, E. S., M.B. | Research Assistant in Pathology. | — |
| Moyle, I. R., M.R.C.S., D.M.R.D. | Registrar in Diagnostic Radiology. | Registrar, Westminster Hospital. |
| Mitchell, J. P., M.B., F.R.C.S. | Consultant in Genito-Urinary Surgery. | Senior Registrar, Bristol. |
| Partridge, J. P., M.B., F.R.C.S. | Senior Registrar in Surgery. | Registrar, Royal Northe[illegible] Hospital. |
| Roseverne, V., M.B., D.M.R.D. | Registrar in Diagnostic Radiology. | Merthyr and Aberdare Group of Hospitals. |
| Ross, F. G. M., M.B., F.F.R. | Consultant in Diagnostic Radiology. | Senior Registrar, Post-graduate Medical Sch[illegible] |
| Roberts, J. A. Fraser, D.Sc., M.D., F.R.C.P., F.R.C.S.(E). | Consultant in Medical Genetics. | — |
| Ruebner, B., M.B. | Registrar in Pathology. | Demonstrator in Pathol[illegible] Bristol. |
| Sleigh, B. E., Miss, M.B., D.A. | Registrar in Anaesthetics. | Resident Anaesthetist, B[illegible] Royal Infirmary. |
| Watson, P. C., M.B., F.R.C.S. | Senior Registrar in Surgery. | Senior Registrar, South[illegible] (Transfer). |

### *BRISTOL*

| *Name* | *Appointment* | *Formerly* |
|---|---|---|
| Barker, J. C. | Registrar, Chest Diseases. | R.M.O., City Hospital, Gloucester. |
| Cross, E. G. W., M.R.C.S., D.P.M. | Assistant Psychiatrist. | Senior Registrar. |
| Fleming, A. C. | Assistant Psychiatrist, Hortham. | — |
| Fyfe, A. E., M.B., M.R.C.O.G. | Registrar in Obstetrics. | Registrar, St. Helier Ho[illegible]pital, Carshalton. |
| Galla, T., M.B. | Registrar in Psychiatry. | — |
| Gilbert, P. M., Miss, M.B. | Registrar, Blood Transfusion Service. | Locum, same post. |
| Harrison, K. G., Miss, M.B., D.R.C.O.G., D.C.H. | Registrar in Pathology, Frenchay. | S.H.O., Frenchay. |
| Hoffman, J. N., M.B. | Assistant Chest Physician. | Registrar, Bristol. |
| Hulme. A., M.B., F.R.C.S. | Consultant Neuro-Surgeon. | Senior Registrar, Bristol. |
| James, B., Miss | Registrar in Paediatrics. | — |
| Knowles, R. R., M.B. | Registrar in Psychiatry. | — |
| Lukianowicz, N., M.D. (Lwow). | Assistant Psychiatrist. | Registrar in Psychiatry. |
| Linton, C. D., M.B., D.R.C.O.G. | Registrar in Anaesthetics, Southmead. | — |
| Mandelbaum, Lisa E. M.B., M.R.C.P. | Senior Registrar, Chest Diseases. | S.H.O. in Medicine, Southmead. |
| Schwartz, S., M.B., F.R.C.S. | Registrar in Orthopaedics. | Registrar, Manchester. |

| *Name* | *Appointment* | *Formerly* |
|---|---|---|
| SEARGEANT, P. W., M.B., F.R.C.S. | Registrar in Surgery, Cossham–Frenchay. | Locum Registrar, Cossham–Frenchay. |
| SIBTHORPE, E. M., M.D., M.R.C.O.G. | Senior Registrar, Obstetrics, Southmead. | Registrar, Old Church Hospital, Romford. |
| SMITH, K. C. P., M.R.C.S., D.P.M. | Psychiatrist, Child Guidance, Gloucestershire C.C. | — |
| TROTTER, J. K., M.R.C.S. | Registrar in Anaesthetics, Frenchay. | — |
| WALKER, W. L., M.B., D.P.M., D.P.H. | Senior Registrar in Mental Deficiency and Child Psychiatry. | Senior Registrar, Moorhaven Hospital, Plymouth. |
| WELLS, C. E. C., M.B., M.R.C.P. | Registrar in Medicine, Southmead. | S.H.O., Bristol United Hospitals. |

### NORTH GLOUCESTER

| *Name* | *Appointment* | *Formerly* |
|---|---|---|
| ELLIS, R. H., M.D., M.R.C.P. | Consultant in Chest Diseases. | — |
| FINN, D. P., M.B. | Assistant Psychiatrist. | — |
| GREEN, D. L. M.B. (SYDNEY), F.R.C.S. (ENGLAND). | Registrar in General Surgery. | — |
| HART, R. J., M.B. | Registrar, Orthopaedics. | — |
| HUNTER, R. M. M., M.B., D.P.M., M.R.C.P. | Consultant Psychiatrist. | — |
| MALLICK, S., B.SC., L.R.C.P. & S., D.R.C.O.G. | Registrar in Obstetrics and Gynaecology. | Registrar in Obstetrics and Gynaecology, Warrington. |
| TOOMEY, A. G. G., B.SC., M.R.C.S., D.M.R.D. | Senior Registrar in Radiology. | Radiological Registrar, United Bristol Hospitals. |

### BATH

| *Name* | *Appointment* | *Formerly* |
|---|---|---|
| SAVILLE, S., M.B. | Registrar, Anaesthetics. | — |
| WHELEHAN, J. M., L.R.C.P., & S.I. | Assistant Psychiatrist. | — |
| YORSTON, R. A., M.B. | Registrar, Surgery of Ear, Nose and Throat. | — |

### EXETER

| *Name* | *Appointment* | *Formerly* |
|---|---|---|
| BOOTHROYD, L. S. A., M.B., F.R.C.S. | Senior Surgical Registrar, Exeter. | — |
| BRIMBLECOMBE, F. S. W. | Consultant Paediatrician, Exeter. | Consultant Paediatrician, Harold Wood Hospital. |
| FISCHER, E., PH.D., L.R., C.P.I. | Assistant Psychiatrist, Exeter (Royal Western Counties Institution). | Registrar, Cell Barnes Hospital. |
| FREEMAN, A. G., M.D., M.R.C.P. | Senior Medical Registrar, Royal Devon and Exeter Hospital. | Assistant Chest Physician Cardiff and District (locum). |

| *Name* | *Appointment* | *Formerly* |
|---|---|---|
| GURRIER, H. P., M.B., F.R.C.S. | Consultant General Surgery, Exeter (Torquay and Newton Abbot). | Senior Surgical Registrar, Guy's Hospital. |
| LARGE, J. M., M.B., F.R.C.S.E. | Senior Registrar, Thoracic Unit, Hawkmoor Chest Hospital. | Senior Registrar, Exeter |
| MINOGUE, F., M.B. | Assistant Psychiatrist (Royal Western Counties Institution). | — |
| SZUR, Z. L., M.B., D.M.R.D., D.M.R. | Assistant Radiotherapist, Exeter. | — |

### *SOUTH SOMERSET*

| *Name* | *Appointment* | *Formerly* |
|---|---|---|
| BOULLE, J. R., M.B. | Registrar, General Surgery, Taunton and Somerset Hospital. | Surgical Registrar, Barry Accident and Surgical Hospital. |
| SCOTT BROWN, J., D.T.M. & H., D.M.R.D. | Consultant, Radiology, South Somerset. | Senior Specialist (Radiolo[illegible] Colonial Medical Ser[illegible] |

### *PLYMOUTH*

| *Name* | *Appointment* | *Formerly* |
|---|---|---|
| ANYAEGBUNAM, P. A. OYE, M.B., D.L.O. | Registrar, Surgery of Ear, Nose and Throat, South Devon and East Cornwall Hospital. | Locum E.N.T. Registrar, Plymouth. |
| HALL, T. E., M.R.C.S. | Registrar, Psychiatry, Moorhaven. | J.H.M.O., Fulbourne Hospital. |
| HEWITT, S. R., M.B., M.R.C.O.G. | Senior Registrar, South Devon and East Cornwall Hospital, Plymouth. | Locum Consultant Gynae[illegible]ogist, Fleet and Dist[illegible] and Yateley and Dist[illegible] Hospitals. |
| PATON, S. L. | Registrar, Mount Gold Orthopaedic Hospital, Plymouth. | — |
| RITCHIE, E. A., M.B., M.R.C.P. | Registrar, Psychiatry, Moorhaven Hospital. | — |
| SMITH, J. A., M.B., F.F.A. | Consultant Anaesthetist, Plymouth. | Lecturer in Anaesthetics, Postgraduate Medical School of London. |
| SMITH, W. H., M.B., D.M.R.D. | Consultant Radiologist, Plymouth. | — |

### *WEST CORNWALL*

| *Name* | *Appointment* | *Formerly* |
|---|---|---|
| ALLISON, R. C., M.B., D.A. | Assistant Anaesthetist, West Cornwall. | G.P. Anaesthetist, Chest[illegible]field Hospital Group. |
| HUGHES, W., M.D., M.R.C.P. | Assistant Physician, Diseases of the Chest, West Cornwall (Resident Physician, Tehidy Chest Hospital). | — |
| MOFFOOT, A. G., MR., M.B., F.R.C.S. | Registrar, General Surgery, Royal Cornwall Infirmary, Truro. | — |
| WINGFIELD, H. V., M.R.C.S., F.R.S.C.E. | Consultant in Thoracic Surgery, West Cornwall. | — |

### Notes and News

Professor R. Milnes Walker has been elected to the Council of the Royal College of Surgeons of England and appointed to a Hunterian Professorship. He has also been made a Fellow of University College, London.

Dr. Beryl Corner has been elected F.R.C.P. (London).

Miss C. Rendle Short has been elected F.R.C.O.G.

Mr. G. J. Hadfield has been appointed Arris and Gale Lecturer, Royal College of Surgeons of England.

Dr. E. H. Hare has won the Gaskell Medal and Prize of the Royal Medico-Psychological Association for 1953.

The following have been made Professors at Makerere College, Uganda:

Mr. H. J. Croot (Surgery).

Dr. J. N. P. Davies (Pathology).

Miss Coralie W. Rendle Short (Obstetrics and Gynaecology).

Dr. J. H. Middlemiss has been invited to join the panel of Consultants for the Colonial Medical Service. He visited Africa for three months from March, 1953.

Dr. J. E. Cates has returned from a year in the United States. He held a fellowship at Stanford University, San Francisco, and was working under Dr. J. A. Luetscher on steroids in urine.

Professor Heller led a research team in Uganda, on behalf of the Medical Research Council and the Colonial Office, to investigate " Water Metabolism ". Dr. H. Schnieden was a member of the team.

Dr. J. Gardiner has been awarded a Leverhulme Fellowship and is working with Professor Lennon.

### CORONATION HONOURS

*O.B.E. (Civil)*: R. A. S. Cory, M.B., CH.B. (BRISTOL).

*M.B.E. (Civil)*: Dorothy Delbridge, M.B., CH.B. (BRISTOL) (*née* Staley).

### SCHOLARSHIPS AND PRIZES

*Bristol Royal Hospital Gold Medal*, 1952: Awarded jointly to F. J. Appelbe and N. D. Sanerkin; no award was therefore made of the Silver Medal.

*Beaverbrook Fellowship*: H. Schnieden.

*Markham Skerritt Memorial Prize*: Dr. J. Grayson.

*Carey Coombs Memorial Prize*: Dr. F. G. Bolton.

*Francis Dudley Prize*: Awarded jointly to B. H. Burns, J. H. Chick and S. J. Strong.

*Edward Fawcett Memorial Prize*: Awarded jointly to H. G. Handel, Sara Hosegood and Rachel M. Hulbert.

*Foyle Prize*: Rachel M. Hulbert.

*Thomas Francis Edgeworth and Francis Henry Edgeworth Prize*: S. C. Jordan.

*Richard Clarke Prize in Child Health*: F. J. Appelbe.

*Mackie Pathology Prize*: D. L. Archer.

*Hewer Pathology Prize*: M. B. Gee and M. L. Cox.

*Tibbits Prize*: A. J. Webb.

*David Rayner Prize in Gynaecology*: 1952, M. E. Jackman; 1953, J. P. Bishop.

*Gold Medal in Child Health and Paediatrics*: Mrs. W. J. Coates.

*Crosby Leonard Prize*: J. L. James.

*Lady Haberfield Scholarship*: D. B. Paintin.

*Marshall and Clarke Prize*: D. B. Paintin.

*Russell Cooper Prize*: D. B. Paintin.

*Sanders' Scholarship*: J. D. Jefferies.

*Medical Suple Prize*: J. D. Jefferies.

*Surgical Suple Prize*: A. J. Webb.

*Henry Clarke Prize*: Winifred J. Coates.

*Barrett Roue Prize*: M. J. Betty.

*Augustin Prichard Prize*: A. J. Webb.

### HIGHER DEGREES AND DIPLOMAS

| | |
|---|---|
| *M.D. (Brist.)* | D. H. Johnson (with Distinction)<br>R. Maggs<br>R. H. Owen |
| *M.D. (Birm.)* | R. L. Bishton |
| *M.D. (Cantab.)* | F. J. Y. Wood |
| *M.Sc.* | H. Sosnowick |
| *Ph.D.* | E. J. Field |
| *F.R.C.S. (Eng.)* | R. A. J. Baily<br>Irene D. R. Gregory<br>P. W. Seargeant |
| *M.R.C.P.* | Roy Hill<br>J. D. Sheward |
| *M.R.C.O.G.* | J. C. S. Leverton |
| *D. P. M. (Brist.)* | E. G. W. Cross<br>J. D. Orme |
| *D.P.H. (Brist.)* | M. C. O'Connor<br>G. D. Teague |
| *D.C.H.* | Brenda James<br>H. M. Perera<br>June Smith<br>Joan Walker |
| *D.A.* | Pamella Hellings |

### UNIVERSITY OF BRISTOL

*M.B., Ch.B. (Brist.), June* 1953

WITH SECOND CLASS HONOURS

Coates, Winifred Jean (with Distinction in Medicine and in Child Health)
Grose, Ruth Janet

PASS

Archer, Desmond Leslie

Bain, Adrian Matheson

Brister, Isabel Kate Elizabeth (with Distinction in Public Health)

Brooks, Thomas Arnold Victor

Cook, John Graham

Coppen, Alec James

Crossley, Rex

Davies, Kenneth David

Downes, Peter William Edward

Edwards, Dorcas

Gee, Michael Barnett

Gordon, Wilfred Edward Lawrence

Jefferies, John Dyment

Lalloo, Ranchor Damodar

MacDonald, Archibald Jeffery

McConnell, Doreen Elizabeth

Nicholas, Philip Owen (with Distinction in Public Health).

Pennefather, Marguerite Eleanor

Perry, James Herbert

Price, Harold

Ree, Michael James

Richards, Thomas David

Shipton, Beryl Maude

Skrine, Ruth Lister

Spry, William Bennett

Stevenson, Audrey Beryl

Taylor, Margaret Gwyneth

Ware, John Walter

Webb, Anthony John

IN GROUP II (COMPLETING THE EXAMINATION)

James, Elizabeth Marjorie

### WHERE CAN I LOOK IT UP?

### *Refresher Courses for General Practitioners.*

The articles which appeared in the *British Medical Journal* under the above title from tober 1949 to December 1950 have now been published in book form—London: itish Medical Association, 1952, 25*s*. A wide variety of everyday medical problems is cussed, and a random choice from the fifty-five topics includes such subjects as the nagement of the premature baby, measles, the causes and treatment of eczema, delay the second stage of labour, the failing heart, the management of the epileptic, the treat- nt of varicose veins, and bronchiectasis. Each chapter is short enough to be read in a minutes. As a rule aetiology, symptoms, signs and diagnosis are briefly reviewed; the phasis, however, is on treatment, and detailed instructions are given about established, l a few recent, therapies. Chapters on penicillin, streptomycin and the newer anti- tics describe their mode of action, use and the kind of infection in which they may be ective—information not always readily available elsewhere.

The articles admirably serve the purpose for which they were designed. The book is ractively produced and reasonably priced. It will be of value to practitioners and dents, and to those specialists who like to know what is afoot in the department next or.

### BRISTOL MARRIAGE AND FAMILY GUIDANCE COUNCIL

Medical practitioners in the South-Western Region may like to be reminded that the stol Marriage and Family Guidance Council has a centre at 77 Park Street, Bristol 1. is voluntary body, by private interview, lectures or discussion groups, offers advice to gaged couples on many aspects of marriage and home-making; assists through trained unsellors those whose marriages are in danger of failing; and gives help with all ques- ns connected with parenthood. In four-and-a-half years over fifteen hundred persons ve sought its aid. The Council makes no charge for its services but, as the expenses of Centre are considerable, appeals to those who recognize the merits of its work to come annual subscribers.

